# Biological control potential of a laboratory selected generalist parasitoid versus a co‐evolved specialist parasitoid against the invasive *Drosophila suzukii*

**DOI:** 10.1111/eva.13605

**Published:** 2023-10-12

**Authors:** Oscar Istas, Marianna Szűcs

**Affiliations:** ^1^ Department of Entomology Michigan State University East Lansing Michigan USA

**Keywords:** adaptation to culture, nonreproductive host killing, parasitoid host range evolution, specialist versus generalist parasitoid, spotted wing drosophila

## Abstract

A few generations of laboratory selection can increase the developmental success of native parasitoids on invasive targets. However, for this approach to be used more widely for biological control, we need to understand if the improved performance of native species, achieved under artificial laboratory conditions, translates to improved control in more natural environments. It is also unknown what the biocontrol potential of laboratory selected generalist native parasitoids may be compared to co‐evolved specialists that are typically introduced for biological control of invasive species. To assess how rearing in artificial diet affected host finding ability in natural hosts, we used laboratory selected (adapted) and nonadapted populations of the generalist native parasitoid *Trichopria drosophilae* to parasitize the invasive fly, *Drosophila suzukii* in three different fruit types. In a separate experiment, we compared the effectiveness of adapted and nonadapted populations of *T. drosophilae* in raspberries with a co‐evolved specialist larval parasitoid *Ganaspis brasiliensis* from Asia that was recently approved for release in the USA. More adult parasitoids emerged in each fruit type of the adapted compared to the nonadapted population of *T. drosophilae*. *D. suzukii* emergence rates were reduced on average by 85% by the adapted *T. drosophilae* population indicating that the artificial rearing conditions did not significantly impair the ability of parasitoids to locate and attack hosts in natural hosts. The specialist *G. brasiliensis* had higher adult emergence than the adapted population of *T. drosophilae*; however, both parasitoid species were able to reduce *D. suzukii* populations to the same extent. These results show that despite the lower developmental success of the laboratory selected *T. drosophilae*, they killed the same proportion of *D. suzukii* as *G. brasiliensis* when host choice was restricted. In nature, where host choices are available, specialist and generalist parasitoids will be unlikely to exhibit the same biocontrol potential.

## 1 INTRODUCTION

Invasive species are most effectively controlled in the long term by the introduction of co‐adapted natural enemies from their native ranges. Since the testing and approval of natural enemies for these so‐called classical biological control introductions can take a long time, often decades, and because there has been a steep decline in such programs (Heimpel & Cock, 2018; Van Driesche et al., 2020), alternative approaches are being explored. One such alternative is to use natural enemies that are already present in the introduced ranges (Jarrett et al., 2022; Kruitwagen et al., 2018; Linder et al., 2022). Even though native species often lack adaptations to effectively attack exotic species, an increasing number of studies demonstrate that a few generations of laboratory selection can significantly improve the developmental success of natural enemies on novel hosts (Henry et al., 2008; Hopper et al., 2021; Jarrett et al., 2022; Jones et al., 2015; Linder et al., 2022). Adaptation in the laboratory can be rapid because strong directional selection is imposed as opposed to diffuse selection in the wild that can slow adaptation. Gene flow can also be limited in laboratory between individuals emerging from natal and novel hosts that could potentially impede incipient adaptations in nature (Kawecki & Ebert, 2004; Strauss et al., 2006). The few proof‐of‐concept studies represent the first steps towards developing laboratory selection as a tool for biological control of invasive pests. However, it is largely unknown whether the improved performance measured under artificial laboratory conditions translates to increased attack rate and developmental success of the invasive targets in more natural environments. For a broader adoption of this method to control invasive species, we also need to understand how the biocontrol potential of laboratory‐improved native natural enemies compares to classical biological control agents that are often co‐evolved specialists on the target invasive species.

Insect parasitoids that lay their eggs inside or on the host and whose developing larvae eventually kills the host are the most commonly used natural enemies to control invasive insect pests (Van Driesche et al., 2020). Parasitoids use visual and chemical cues both from the insect host and from the plants their hosts feed on to locate targets on the landscape (Dicke & Baldwin, 2010; Gandolfi et al., 2003; Turlings et al., 1991; Vet et al., 1995; Vet & Dicke, 1992). However, during laboratory rearing, these cues can be largely absent or significantly altered. For example, for the rearing of drosophilid flies that are the subject of our investigations, a cornmeal, yeast and sugar based artificial diet is used instead of fruit. Parasitoids are then released in small plastic vials with artificial diet that contain the developing fly larvae or pupae to be parasitized. To build up populations for mass release or experiments, both the parasitoids and flies spend tens or hundreds of generations in laboratory rearing under these artificial conditions. Such long‐term laboratory rearing was shown to alter courtship behavior, host finding and foraging behaviors in parasitoids, and the artificial rearing substrates can impact parasitoid vibrational communication and host acceptance (Bautista & Harris, 1997; Canale & Benelli, 2012; Gandolfi et al., 2003; Ghaemmaghami et al., 2022; Joyce et al., 2008; Naranjo‐Guevara et al., 2020). The fitness of parasitoids may also be reduced with prolonged laboratory rearing as they adapt to the rearing environment and undergo demographic and genetic changes (Hopper et al., 1993; Nunney, 2003; Szűcs et al., 2019). In cases when the laboratory colony is initiated with few individuals and/or the populations pass through bottlenecks, genetic diversity can be reduced, genetic load can increase via drift, and inbreeding can ensue upon mating between related individuals (Fauvergue et al., 2012; van Lenteren, 2003). Because of the negative effects of prolonged laboratory rearing periodical augmentation using wild caught individuals is recommended to restore fitness of mass reared parasitoids (Bartlett, 2018; Ghaemmaghami et al., 2022; Hoekstra, 2003). However, this may not be a feasible approach when laboratory selection is used to improve certain traits because gene flow between the added wild‐type parasitoids, and the selected individuals could swamp the targeted adaptations (Lenormand, 2002; Tallmon et al., 2004; Whiteley et al., 2015). Thus, it is important to test parasitoids that have undergone selection and long‐term laboratory rearing without augmentation of wild‐type individuals for their ability to find and attack target species that are developing in real host plants.

Classical biological control introductions most often use specialist endoparasitoids (Van Driesche et al., 2020). Development inside a host requires unique adaptations to overcome host defenses (Godfray, 1994). These specialized adaptations usually make the parasitoids quite host specific, which is a necessary component of classical biological control programs to avoid nontarget effects on native species (Hajek & Eilenberg, 2018; Heimpel & Mills, 2017). Highly specialized parasitoids also tend to have fine‐tuned adaptations to locate the narrow range of hosts they are able to exploit (Godfray, 1994). These traits can make specialist parasitoids very efficient biocontrol agents with relatively high attack rates of the target species (Godfray, 1994; Hajek & Eilenberg, 2018; Heimpel & Mills, 2017; Van Driesche et al., 2020). However, when an exotic host invades a novel habitat, generalist parasitoids are usually the first to attack them (Abram et al., 2019; Chris Gröbler & Lewis, 2008; Cornell & Hawkins, 1993; Grabenweger et al., 2010; Lee et al., 2019), and it is usually generalist parasitoids that respond rapidly to selection on novel hosts (Golec et al., 2019; Henry et al., 2008; Jarrett et al., 2022; Jones et al., 2015; Linder et al., 2022). Even though generalists, by definition, are adapted to attack a wide range of species, their parasitism rates can vary widely both on native and exotic hosts (Chris Gröbler & Lewis, 2008; Cornell & Hawkins, 1993; Grabenweger et al., 2010). Attack rates of exotic hosts by parasitoid communities dominated by generalist species in the introduced range tend to be lower than parasitism rates in their native ranges where they are attacked by a combination of specialist and generalist parasitoids (Cornell & Hawkins, 1993; Grabenweger et al., 2010; but see Vindstad et al., 2013). It is unknown how the performance of an experimentally improved generalist parasitoid would compare to a co‐evolved specialist parasitoid from the native range of exotic species.

The focal pest of this study is spotted wing drosophila (*Drosophila suzukii*) (Matsumura) (Diptera: Drosophilidae), a polyphagous pest of soft fruits, native to Southeast Asia that invaded Europe and continental North America in 2008 (Asplen et al., 2015; Lee et al., 2019). In the introduced range of the United States, only two generalist pupal parasitoids, *Pachycrepoideus vindemmiae* (Rondani) (Hymenoptera: Pteromalidae) and *Trichopria drosophilae* (Perkins) (Hymenoptera: Diapriidae), are known to attack *D. suzukii* at low levels (<10% parasitism rates) (Lee et al., 2019). Previous studies showed that laboratory selection could significantly increase the developmental success of these two parasitoid species on *D. suzukii* (Jarrett et al., 2022; Linder et al., 2022). Recently, a classical biological control agent, the specialist larval parasitoid, *Ganaspis brasiliensis* (Ihering) (Hymenoptera: Figitidae) was approved for field releases in North America against *D. suzukii* (Daane et al., 2021).

Here, we explore whether a laboratory selected population of *T. drosophilae* also shows increased attack rates of *D. suzukii* in more natural environments by comparing their performance with that of nonadapted *T. drosophilae* in three different fruit types (raspberries, cherries, and blueberries). We also assess how release density influences parasitism and how prolonged laboratory rearing may have affected foraging behavior by testing parasitism rates of fruit that is either placed on the ground (i.e., fallen fruit) or elevated (i.e., fruit on the plant). In addition, we compare the biocontrol potential of laboratory improved generalist parasitoids and of a specialist biocontrol agent by evaluating their parasitism rates on raspberry plants. We hypothesize that the laboratory rearing, and selection process did not impair the ability of parasitoids to locate and attack *D. suzukii* in fruit. We expected that the adapted population of *T. drosophilae* would continue to exhibit higher attack rates in all fruit types compared to the nonadapted *T. drosophilae* population regardless of parasitoid density and the placement of fruit. We expected *G. brasiliensis* to be a superior biocontrol agent compared to the adapted population of *T. drosophilae* given that it is a co‐evolved specialist while the latter is a generalist that had been subject to 10 generations of laboratory selection.

## 2 MATERIALS AND METHODS

### 2.1 Fly colonies

The origin and rearing procedures of the *D. suzukii* and *D. melanogaster* colonies were described in Jarrett et al. (2022). Drosophila melanogaster was used as the control host during the selection experiments and later for rearing the adapted and nonadapted populations of *T. drosophilae* (see below). Briefly, the flies were reared using a standard DSSC cornmeal diet at 25 ± 2°C, 16L:8D photoperiod, and at 85 ± 5% humidity. Population sizes of >1000 individuals were maintained in standard drosophila vials of 2.5 × 9.5 cm (Genesee Scientific, San Diego, CA, USA) at densities of around 30 flies per vial.

### 2.2 Parasitoid colonies

*Trichopria drosophilae* is a solitary, cosmopolitan pupal parasitoid of flies in the Drosophilidae family (Carton et al., 1986). Our laboratory colony was founded by 30 individuals from a North American population as described in Jarrett et al. (2022). Three replicate populations of *T. drosophilae* were reared for 10 generations on *D. suzukii* as part of previous selection experiments, and three populations were reared on *D. melanogaster* representing the control treatment for those experiments (Jarrett et al., 2022; Linder et al., 2022). The populations that responded positively to selection for increased developmental success on *D. suzukii* in these prior experiments will be referred to as ‘adapted’ or ‘improved’ and the controls as ‘nonadapted’ populations. As the selection experiments concluded, the adapted populations of *T. drosophilae* were moved back onto the ancestral host, *D. melanogaster* for rearing for five generations for ease of maintenance. Two generations prior to the start of the current experiments, the three replicate adapted *T. drosophilae* populations were moved back onto the invasive host, *D. suzukii*, while the three nonadapted populations remained on *D. melanogaster*. Parasitoids were reared by releasing ~25 adults in vials that contained 5‐day‐old pupae of the respective fly species. Parasitoids were provided with a strip of paper towel infused with honey and left in the vials for 48 h to parasitize fly pupae. Adult parasitoids emerged within 5 weeks and were either used for experiments or to maintain colonies.

*Ganaspis brasiliensis* is an Asian solitary larval parasitoid (Daane et al., 2021; Wang et al., 2020). An East Asian strain (G1) of *G. brasiliensis* has a narrow host range that is restricted to a few species within the genus *Drosophila* and had been approved for field releases in the continental USA as a classical biological control agent against *D. suzukii* (Daane et al., 2021; Hougardy et al., 2022; Wang et al., 2020). A colony of *G. brasiliensis* was initiated at Michigan State University (MSU) using 50 female and 50 male adults from a USDA‐APHIS laboratory in Newark, NJ. The *G. brasiliensis* colony used in the experiments described below were initiated by 15 females and 15 males from the MSU core colony. To rear *G. brasiliensis* store‐bought, conventionally grown blueberries were infested by *D. suzukii* at densities of 100 flies per 300 grams of fruit in clear plastic containers (25 × 19 × 25 cm; PrepNaturals, Philadelphia, PA, USA). Berries were lightly dusted by instant dry yeast (Fleischmann's ActiveDry yeast) to increase attraction of flies and to prevent mold formation during the rearing process (Rossi‐Stacconi et al., 2022). Two days following infestation when the fly larvae were in the first instar stages, 30 *G. brasiliensis* adults were released into the containers with the *D. suzukii* for 5 days for parasitization. Adults were reused to parasitize infested fruit up to three times. The next generation of parasitoids emerged in 5 weeks. The rearing took place in the same incubators used for fly rearing using the same temperature and light settings.

### 2.3 Experiment 1 – Testing the effectiveness of adapted versus nonadapted populations of *Trichopria drosophilae* in fruit

Two generations after the three replicate adapted populations were moved back from *D. melanogaster* onto *D. suzukii* for rearing we retested their developmental success on the invasive host. For this test, 20 female parasitoids were drawn randomly from each of the three replicated adapted and nonadapted populations. Each female parasitoid was provided with 10 *D. suzukii* pupae for 48 h to parasitize. The pupae were removed from the artificial diet used for fly rearing and presented in a clear Petri dish (60 × 15 mm; Falcon, Corning, NY, USA) for parasitization. The numbers of emerging flies and parasitoids were monitored for 5 weeks. This test confirmed that differences in development success between the selected and control lines on *D. suzukii* were maintained despite the five generations of rearing of both on *D. melanogaster* (Figures S1 and S2). Following this test, the three replicate selection populations were merged into a single adapted population for further experimentation. The same was done for the three replicate nonadapted populations, resulting in one adapted and one nonadapted *T. drosophilae* populations for experimentation.

The performance of the adapted and nonadapted population (selection treatments) of *T. drosophilae* was compared in three types of fruit (raspberries, sweet cherries, and blueberries) placed at two different heights (on the ground or elevated) at two different parasitoid densities (50 or 100). There were eight replicates for each selection treatment for each fruit type and for each parasitoid densities tested at two heights (2 selection treatments × 8 replicates × 3 fruit types × 2 parasitoid densities × 2 heights = 192 trials). In addition, there were eight replications that were used to test *D. suzukii* emergence success from each fruit type without any parasitism (controls). The eight replications for each treatment combination were split evenly between two temporal blocks in two consecutive weeks for logistical reasons. Each fruit type tested was store bought and organically grown. After a thorough wash, 50 grams of fruit were placed in plastic containers (25 × 19 × 25 cm, PrepNaturals) and dusted lightly with yeast (see rearing methods for *G. brasiliensis*). These containers were then placed at the bottom of mesh cages (30 × 30 × 30 cm, Restcloud) with two containers per cage (per replicate) on a tabletop in the laboratory at ambient temperature (22–25°C). In each cage, 100 *D. suzukii* adults were released to infest the fruit for 48 h. Flies were provided with honey water via a cotton wick placed in a 59 mL cup during infestation. After 2 days the flies were removed, and each plastic container was fitted with a lid that had a 12 × 8 cm square mesh screen for ventilation. The containers with the infested fruit were then placed into the incubators used for fly rearing for 72 h to allow the fly larvae to mature into pupae. These containers were then placed back into the mesh cages used to infest them with flies. In each cage, one of the containers was placed on the ground and the other container was suspended 20 cm from the bottom of the cage using plastic supports secured to the mesh cage walls. The cages then received either 50 or 100 *T. drosophilae* adult parasitoids from either the adapted or nonadapted populations or no parasitoids. After 48 h of parasitism, the containers with the fruit were removed from the cages and labeled, noting the replication number, selection treatment, parasitoid density treatment and the location during parasitism. They were fitted with lids with fine mesh squares and placed into the rearing incubators. Twice a week for 5 weeks, the numbers of emerging flies and parasitoids in each container were recorded and the insects were removed from the containers.

### 2.4 Experiment 2 – Testing the effectiveness of adapted populations of *Trichopria drosophila* versus the specialist *Ganaspis brasiliensis* on raspberry plants

To compare the performance of laboratory selected populations of the generalist *T. drosophilae* and the specialist *G. brasiliensis*, raspberry plants were introduced alongside the fruit to provide more cues related to foraging for hosts than the fruit tested in the previous experiment could provide on its own. Parasitism levels of three different parasitoid treatments (adapted and nonadapted *T. drosophilae*, and *G. brasiliensis*) were tested on one fruit type (raspberry) that was placed on both the ground and elevated to the fruiting level of the raspberry plant to offer a choice in foraging height. There were 12 replications for each parasitoid treatment and 12 additional replications were used to test emergence success of *D. suzukii* from fruit not exposed to parasitism. In April 2022, 1‐year‐old bare root ‘Heritage’ raspberries (*Rubus idaeus*, Indiana Berry and Plant Co.) were planted into 8.517 L plastic pots using Suremix Perlite potting mix (Cleveland, OH, USA). The potted plants were kept in the greenhouse until mid‐May when they were taken outside and dug into the soil at the MSU Entomology farm up to the rim of the pots. In late September the plants with the pots were lifted from the ground and used for experiments. At this point, plants were 40–60 cm tall with some fruit and flowers. Since the amount of fruit differed among plants, we removed the berries and standardized the amount of store‐bought raspberries in the experiment.

The potted plants were placed individually into tall mesh cages (61 × 40 × 40 cm, Restcloud) in a greenhouse at 22°C. Infestation of raspberries proceeded as described for experiment 1 by placing 50 grams of fruit into plastic containers and releasing 100 *D. suzukii* individuals in each cage to lay eggs for 48 h. However, given that *T. drosophilae* is a pupal parasitoid and *G. brasiliensis* is a larval parasitoid, the infested fruit was not incubated prior to the release of *G. brasiliensis* but was incubated for 3 days prior to the release of *T. drosophilae* to reach the pupal stage. In each cage, one of the containers with the infested fruit was placed on the bottom of the cage, and the other container was elevated to the canopy level of the raspberry plants. Fifty parasitoids of the given treatments were released in each cage and left to parasitize for 48 h. The containers with the fruit were then removed from the cages and placed into the rearing incubators where *D. suzukii* and parasitoid emergence was checked twice a week for 5 weeks.

### 2.5 Statistical methods

All analyses were conducted using R software 4.2.3 (R Core Team, 2022). Generalized linear mixed effects models (glmer) were constructed in the *lme4* package, and the ANOVA method was used to compute test statistics (Bates et al., 2015). Histograms and the Shapiro–Wilk test were used to check the distribution of the data. Data for both experiments were analyzed using a negative binomial distribution to account for overdispersion. Akaike Information Criterion (AIC) was used to select the model with the best fit by comparing the simplest model without any interactions to models with all possible combinations of two‐way and three‐way interactions. All post hoc pairwise comparisons were performed using the *emmeans* package with Tukey adjustment (Lenth et al., 2021).

For the first experiment, the number of emerging parasitoids was compared by including selection treatment (adapted vs. nonadapted *T. drosophilae*), fruit type (raspberry, cherry, or blueberry), parasitoid density (50 or 100), the position of fruit during parasitism (ground vs. elevated) and the interactions between fruit type and wasp density, and fruit type and fruit position as fixed effects. Temporal block was included as a random effect. To assess the effect of different parasitoid treatments on the number of flies emerging, we included the control treatment with no parasitoid release in the analysis and compared this to the treatments that used adapted and nonadapted parasitoid releases. Parasitoid treatment and fruit type were fixed effects and temporal block was a random effect in the model.

For the second experiment, to compare the number of parasitoids emerging, the three parasitoid treatments (adapted vs. nonadapted *T. drosophilae* and *G. brasiliensis*) and the position of fruit during parasitism (ground vs. elevated) were the main factors in the model. To test how parasitism affected fly emergence, the three parasitoid treatments were included as main factors in the model.

## 3 RESULTS

### 3.1 Experiment 1 – Testing the effectiveness of adapted versus nonadapted populations of *Trichopria drosophilae* in fruit

**FIGURE 1:**
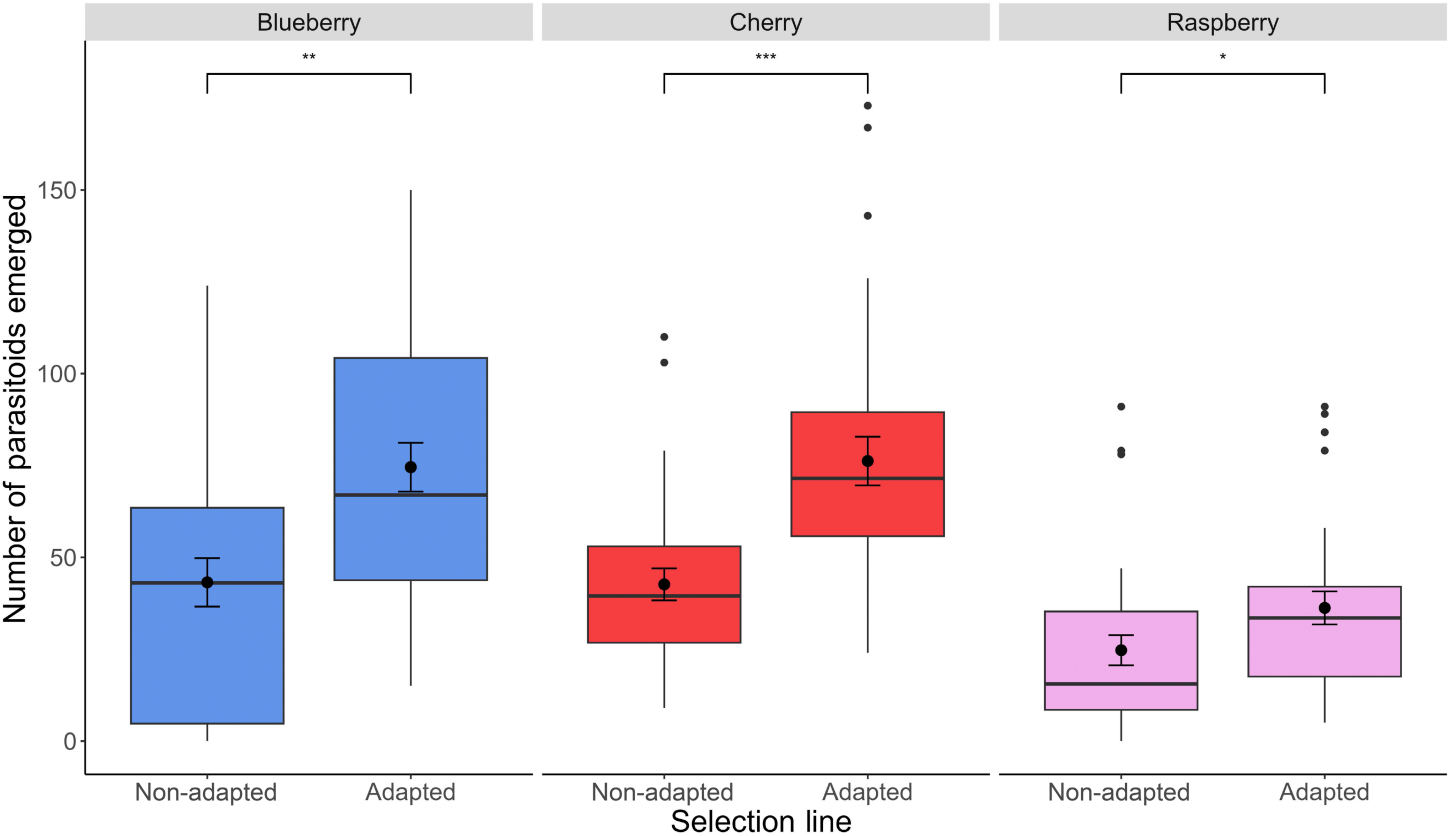
The number of *Trichopria drosophilae* adult parasitoids emerging from three types of fruit (blueberries – blue boxes, sweet cherries – red boxes, and raspberries – pink boxes) infested by *Drosophila suzukii*. *Trichopria drosophilae* populations had previously been either selected (Adapted) or had not been selected (non‐Adapted) for improved developmental success on *D. suzukii*. Dots indicate outlier observations, the horizontal line indicates the median with the box representing the interquartile range, and vertical lines are 1.5 times the interquartile range. Means and standard errors are shown within each box plot. Asterisks indicate significant differences between treatments at the 0.5 (*), 0.05 (**), and 0.005 (***) levels.

**FIGURE 2:**
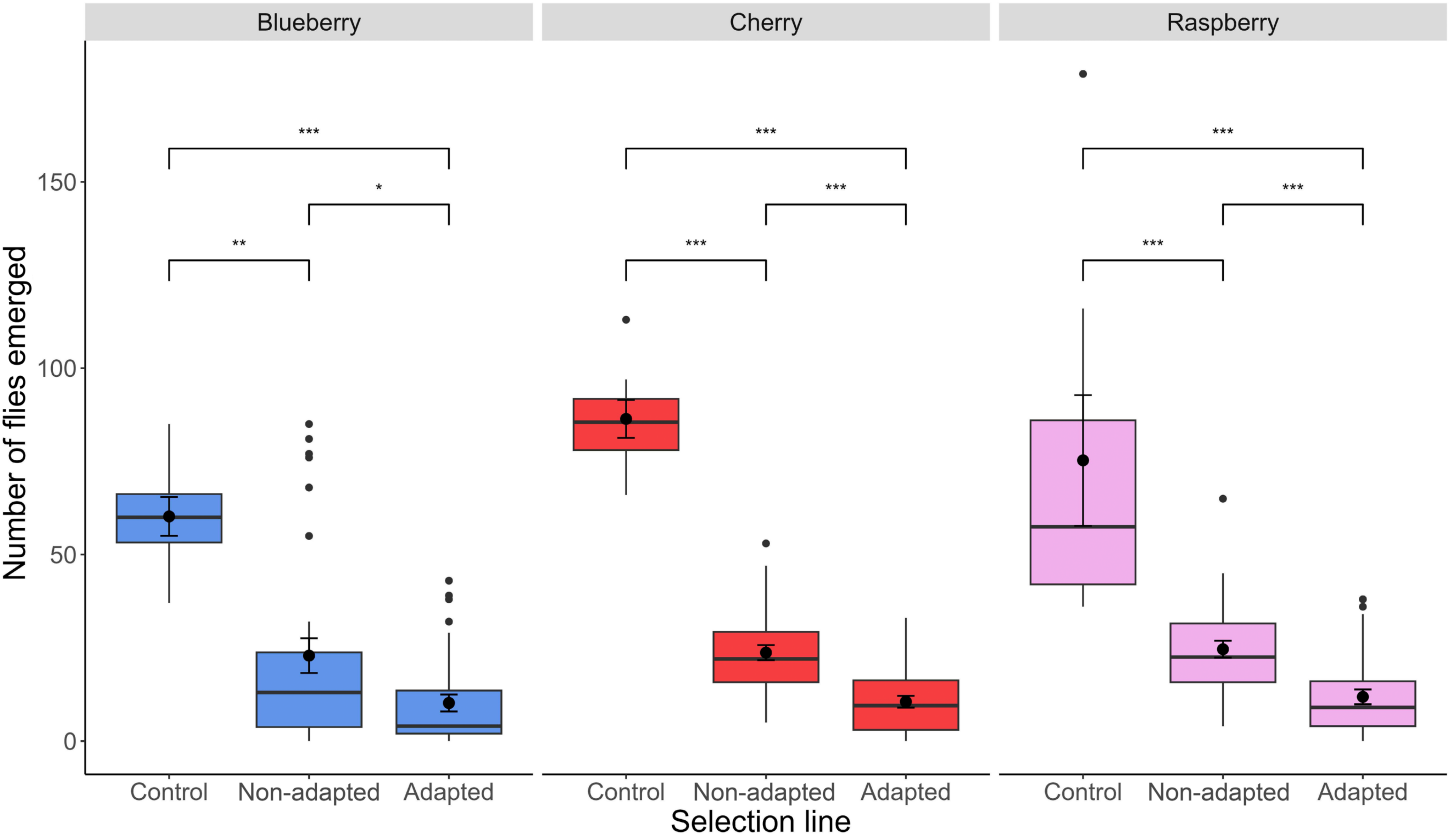
The number of *Drosophila suzukii* adult flies emerging from three types of fruit (blueberries – blue boxes, sweet cherries – red boxes, and raspberries – pink boxes). Infested fruit was either not attacked by parasitoids (Control) or were attacked by *Trichopria drosophilae* that had previously been selected (Adapted) or had not been selected (non‐Adapted) for improved developmental success on *D. suzukii*. Dots indicate outlier observations, the horizontal line indicates the median with the box representing the interquartile range, and vertical lines are 1.5 times the interquartile range. Means and standard errors are shown within each box plot. Asterisks indicate significant differences between treatments at the 0.5 (*), 0.05 (**), and 0.005 (***) levels.

The number of parasitoids emerging of the adapted and nonadapted *T. drosophilae* populations from *D. suzukii*‐infested fruit was significantly different (*χ* ^2^ = 25.70; *df* = 1, *p* < 0.0001). The number of parasitoids emerging from all fruit types was higher when attacked by the adapted compared to the nonadapted population of *T. drosophilae* (all pairwise comparisons *p* < 0.0001) (Figure 1). Neither the placement of fruit on or off ground (*χ* ^2^ = 1.58, *df* = 1, *p* = 0.21) nor the number of parasitoids released (*χ* ^2^ = 0.04, *df* = 1, *p* = 0.84) affected parasitoid emergence. The number of *D. suzukii* emerging differed among parasitoid treatments (*χ* ^2^ = 110.70, *df* = 2, *p* < 0.0001) but not among the different fruit types (*χ* ^2^ = 0.74, *df* = 2, *p* = 0.69) (Figure 2). The greatest number of flies emerged from the control treatment (73.7 ± 12.44; mean ± SE) that received no parasitoids. Parasitism by the nonadapted *T. drosophilae* population reduced fly emergence by 68% (23.47 ± 2.04), and parasitism by the adapted population reduced fly emergence by 85% (10.80 ± 0.97) (all pairwise comparisons are significant at <0.05) (Figure 2).

### 3.2 Experiment 2 – Testing the effectiveness of adapted populations of *Trichopria drosophila* versus the specialist *Ganaspis brasiliensis* with live plants

**FIGURE 3:**
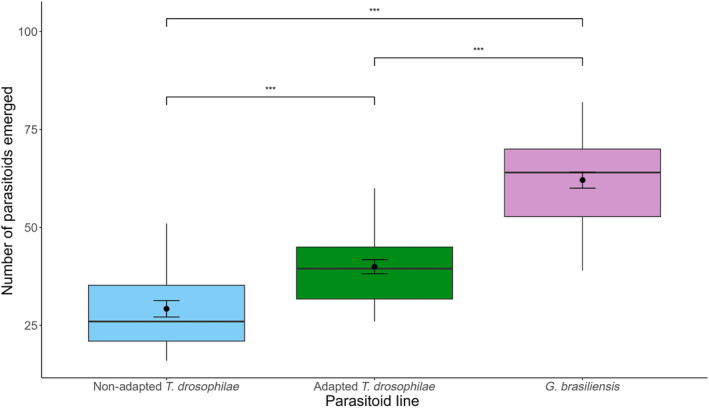
The number of *Trichopria drosophilae* or *Ganaspis brasiliensis* adult parasitoids emerging from raspberries infested by *Drosophila suzukii*. *Trichopria drosophilae* populations had previously been either selected (adapted) or had not been selected (nonadapted) for improved developmental success on *D. suzukii*. The horizontal line indicates the median with the box representing the interquartile range, and vertical lines are 1.5 times the interquartile range. Means and standard errors are shown within each box plot. Asterisks indicate significant differences between treatments at 0.005 (***) levels.

**FIGURE 4:**
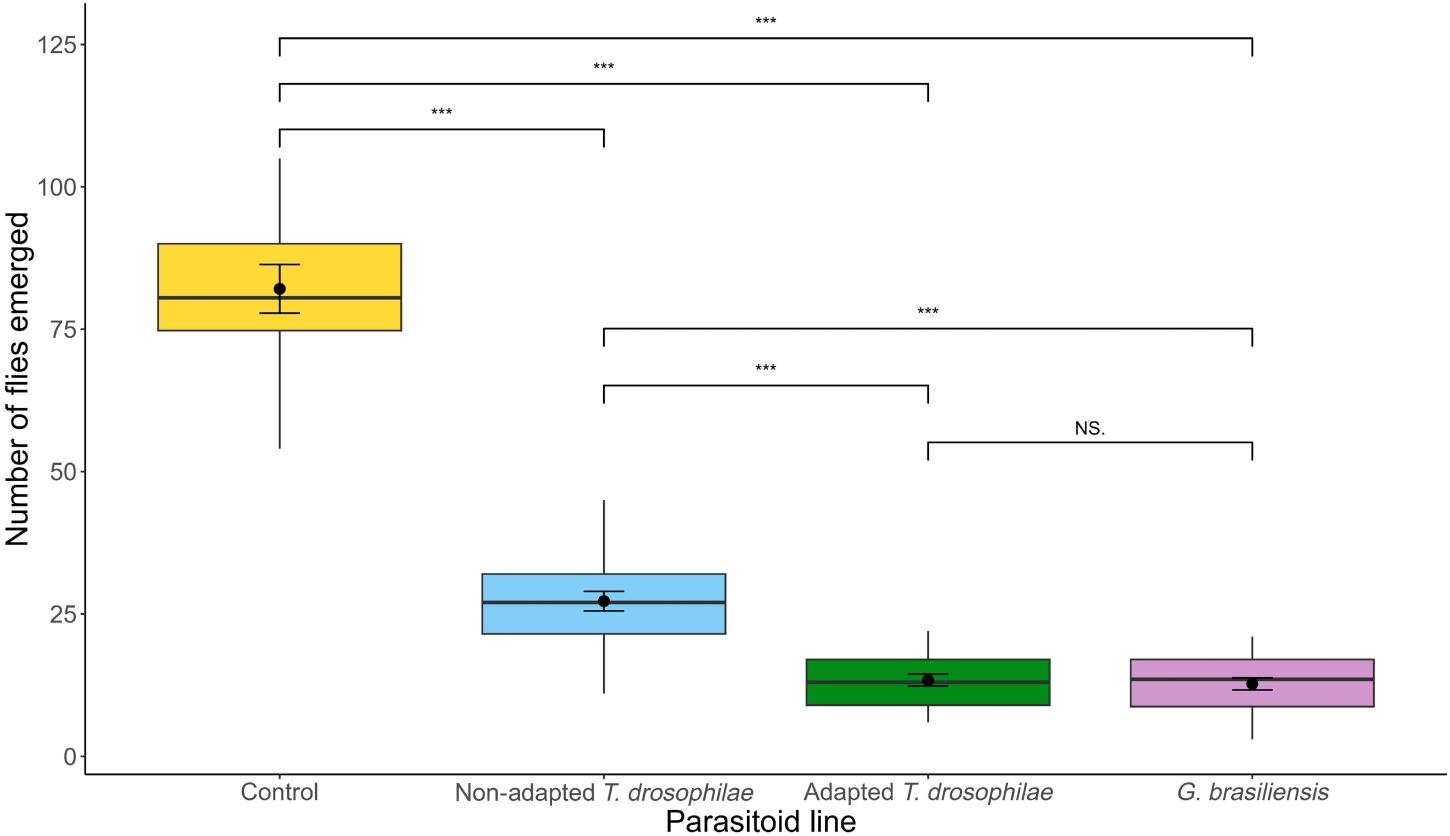
The number of *Drosophila suzukii* adult flies emerging from raspberries. Infested raspberries were either not attacked by parasitoids (Control) or were attacked by three different parasitoid populations: (1) *Trichopria drosophilae* that had previously been selected (Adapted) (2) or had not been selected (non‐Adapted) for improved developmental success on *D. suzukii*, and (3) *Ganaspis brasiliensis* that is a specialist larval parasitoid. Dots indicate outlier observations, the horizontal line indicates the median with the box representing the interquartile range, and vertical lines are 1.5 times the interquartile range. Means and standard errors are shown within each box plot. Asterisks indicate significant differences between treatments at 0.005 (***) levels.

The number of emerging parasitoids from *D. suzukii* infested raspberries differed among the three parasitoid treatments (*χ* ^2^ = 130.79, *df* = 2, *p* < 0.0001). The specialist *G. brasiliensis* emerged at the highest numbers (62.1 ± 2.71; mean ± SE), followed by the adapted population of *T. drosophilae* (40.0 ± 1.91), with the nonadapted *T. drosophilae* population yielding the fewest parasitoids (29.2 ± 1.51) (all pairwise comparisons are significant at <0.05) (Figure 3). The placement of the fruit on or off ground had no effect on parasitoid emergence (*χ* ^2^ = 0.24, *df* = 1, *p* = 0.63). The number of emerging flies was significantly different between parasitoid treatments (*χ* ^2^ = 482.48, *df* = 3, *p* < 0.0001). Most flies emerged from the control treatment (82.1 ± 6.03) that received no parasitoids (Figure 4). Parasitism by the nonadapted population of *T. drosophilae* reduced fly emergence by 67% (27.2 ± 1.66) (pairwise comparison: *p* < 0.0001). Interestingly, the number of emerging *D. suzukii* was similar for *G. brasiliensis* (12.7 ± 0.94) and the adapted population of *T. drosophilae* (13.4 ± 0.97) (pairwise comparison: *p* = 0.96). Attack by either of these two parasitoid populations reduced fly emergence by 84%–85% compared to the controls (both pairwise comparisons are <0.05) (Figure 4).

## 4 DISCUSSION

We found that the *T. drosophilae* population that was selected in the laboratory for improved performance on the invasive *D. suzukii* showed increased biocontrol efficacy compared to the nonadapted *T. drosophila* population under more realistic conditions, including in multiple different types of fruit and when fruit was offered alongside a live plant. This improved parasitism success has been maintained despite rearing of the adapted *T. drosophila* population on a native host, *D. melanogaster*, for several generations following 10 generations of selection on the invasive host. As expected, we also found that the co‐evolved specialist parasitoid *G. brasiliensis* had higher adult emergence success from *D. suzukii* than the laboratory improved population of *T. drosophilae*. Surprisingly, these two parasitoid populations were able to reduce emerging *D. suzukii* numbers to the same extent via the combination of increased reproductive success and nonreproductive host killing by *T. drosophilae*.

One of the main concerns during laboratory rearing is behavioral changes in parasitoids including the deterioration of host searching and oviposition behaviors especially when artificial rearing substrates are used (Gandolfi et al., 2003; Lewis & Tumlinson, 1988; van Lenteren, 2003; Vet et al., 1995; Vet & Dicke, 1992). For example, the fruit fly parasitoid *Diachasmimorpha longicaudata* (Ashmead) that was reared for over 160 generations on oriental fruit flies (*Bactrocera dorsalis* Hendel) raised on a semi‐synthetic wheat diet preferred to oviposit in flies that developed in the wheat diet compared to flies that developed in papaya (Bautista & Harris, 1997). Rearing of codling moth (*Cydia pomonella* L.) on artificial diet for 30 generations also changed the host searching behavior of the larval parasitoid *Hyssopus pallidus* compared to when the moth host was raised on a natural apple diet (Gandolfi et al., 2003). The frass produced by moth larvae while feeding on apples serves as a cue to locate hosts in nature but the parasitoids that were reared under artificial conditions had a 53% lower search response to this cue than those reared in the natural system with apples (Gandolfi et al., 2003). Yet, we found that *T. drosophilae* has maintained its ability to locate *D. suzukii* in multiple types of fruit despite the long‐term laboratory rearing during which hosts were offered in an artificial diet. The foraging behavior of parasitoids did not change either as they were able to locate hosts on the ground and at elevated positions similarly as they forage in nature (Rossi Stacconi et al., 2019). There is some evidence that *T. drosophilae* may prefer to parasitize fly pupae on the ground (Wolf et al., 2021) but in our experiments there was no difference in parasitoid emergence from elevated fruit and those placed on the ground. On average across different fruits the improved *T. drosophila* population reduced *D. suzukii* emergence by 85% (Figure 2). When a raspberry plant was present to provide additional foraging cues, the adapted *T. drosophilae* population and the specialist *G. brasiliensis* both reduced fly emergence by 84%–85% (Figure 4). These results are encouraging regarding the potential effectiveness of laboratory improved parasitoids for release in the wild.

Previous experimental results have shown that changes in parasitoid behavior in artificial rearing environments may be transient plastic responses that can be reversed or improved rapidly with learning upon exposure to natural plant‐related cues (Gandolfi et al., 2003; Lewis & Tumlinson, 1988; Vet & Dicke, 1992). In the codling moth system, for example, *H. pallidus* parasitoids quickly learned to associate cues from apples with the presence of hosts and exposure to apple cues during development or as early adults for a single generation was enough to revert the deterioration in their host searching behaviors (Gandolfi et al., 2003). While plant‐related cues may be plastic traits, host‐related cues are more reliable from a parasitoid's perspective, and they are likely to be genetically based (Lewis & Tumlinson, 1988; Vet & Dicke, 1992). Therefore, prolonged exposure to altered host cues could be subject to rapid evolution, for example, in cases when factitious hosts are used during laboratory rearing (Bourchier et al., 1993; Gowda et al., 2021; van Lenteren, 2003). However, if the target host is used for rearing, the foraging potential of laboratory reared parasitoids should not be compromised significantly.

The adapted population of *T. drosophilae* had consistently higher emergence rates in each tested fruit compared to the nonadapted population (Figures 1 and 3). Thus, adaptation to *D. suzukii* has been maintained despite multiple generations of rearing on their ancestral host *D. melanogaster* in between the end of the original selection experiment (Jarrett et al., 2022; Linder et al., 2022) and the beginning of the fruit assays. Attack rates of *D. suzukii* were high across all types of fruit (Figures 2 and 4), and the number of parasitoids emerging was relatively high in blueberries and cherries considering the number of flies available for parasitism (Figures 1 and 3). These results indicate that the fitness of parasitoid populations has not declined during long‐term laboratory rearing. Alternatively, the high attack rates (68%–85% killing efficiency) and the relatively high developmental success of both the adapted and nonadapted *T. drosophilae* population could be because we combined the three replicate selection populations into a single population and did the same for the three control populations before testing them on the different fruits. The merging of populations may have resulted in both demographic and genetic rescue as it increased population sizes, has likely alleviated genetic load, and mating between individuals that may have had disparate adaptations to the new host could have created new, successful variants (Hufbauer et al., 2015; Tallmon et al., 2004; Whiteley et al., 2015). Similar approaches have been recommended to maintain parasitoid fitness during mass rearing that include the maintenance of isofemale inbred lines that are merged prior to release. Another approach uses compartmentalization where the population is separated into multiple vials that are combined at set intervals to facilitate interbreeding among lineages (Bartlett, 2018; Nunney, 2003; van de Zande et al., 2014). Recent experimental research in Trinidadian guppies (*Poecilia reticulata*) shows that gene flow does not necessarily overwhelm local adaptive traits (Fitzpatrick et al., 2020). Therefore, introduction of a few field‐collected individuals into a laboratory colony may not erase host adaptation. However, it seems that fitness may be preserved without adding wild‐type individuals via the maintenance of isolated replicate populations that could be used to create outbreeding events.

In terms of adult emergence, the newly approved biological control agent *G. brasiliensis* was more successful than the laboratory improved population of *T. drosophilae*. These results are not surprising given that the strain of *G. brasiliensis* that is approved for field releases in the USA and was used in our experiments is a co‐evolved specialist on *D. suzukii* (Daane et al., 2021; Hougardy et al., 2022). We also know from previous studies that despite substantial improvement of developmental success in response to laboratory selection, *T. drosophilae* was not able to reach similar emergence rates on *D. suzukii* than another generalist pupal parasitoid, *P. vindemmiae* (Jarrett et al., 2022; Linder et al., 2022). This was likely because *T. drosophilae* is an endoparasitoid and *P. vindemmiae* is an ectoparasitoid (Wang et al., 2020). Endoparasitoids need adaptations such as specialized venoms to overcome host defenses since they develop inside the host (Godfray, 1994; Wang et al., 2016, 2020). Even though it is difficult to directly compare larval and pupal parasitoids, both *G. brasiliensis* and *T. drosophilae* are endoparasitoids and thus need adaptations to contend with host defenses. It is likely that *G. brasiliensis*, with its long‐term co‐evolutionary history, is more effective at overcoming *D. suzukii* host defenses than *T. drosophilae* that was subject to relatively short‐term selection to improve its development success on this novel host.

However, developmental success is not the only measure to assess biocontrol potential of parasitoids. One of the potential outcomes of parasitism is the death of hosts without the successful development of parasitoids (Abram et al., 2016, 2019). This so‐called nonreproductive host killing happens often when native parasitoids try to attack novel invasive hosts that they are not necessarily able to develop on (Abram et al., 2016, 2019; Kaser et al., 2018; Kruitwagen et al., 2021). Nonreproductive host killing is a heritable trait that can respond to selection (Kruitwagen et al., 2021). When the native larval parasitoid, *Leptopilina heterotoma* (Hymenoptera: Figitidae) was subjected to laboratory selection, its killing rate of *D. suzukii* improved somewhat but not its developmental success (Kruitwagen et al., 2021). We found that *G. brasiliensis* and the improved population of *T. drosophilae* reduced *D. suzukii* emergence to the same extent (Figure 4). This indicates that, although fewer *T. drosophilae* successfully developed and emerged on *D. suzukii*, they killed the same proportion of flies as the co‐evolved *G. brasiliensis*. The increased killing rate that is detected between the adapted and nonadapted population of *T. drosophilae* on the different types of fruit (Figures 3 and 4) could be a correlated response to selection for improved developmental success on *D. suzukii*.

This study demonstrates that improved parasitoid performance on a novel host accomplished in the laboratory using artificial diet can translate to increased attack rates in natural host plants. We also show that in the laboratory under no‐choice conditions a generalist pupal parasitoid selected for improved performance on a novel target can achieve similar biocontrol potential as a specialist larval parasitoid with the combined result of improvements in developmental success and of nonreproductive host killing rate. In nature, generalist parasitoids, such as *T. drosophilae*, have multiple host species to choose from, while specialists like *G. brasiliensis* will preferentially target *D. suzukii*. Nonreproductive host killing would also have a negative impact on *T. drosophilae* demography compared to the higher developmental success and thus higher population growth of *G. brasiliensis*. Therefore, it is unlikely that the observed high biocontrol potential of *T. drosophilae* would translate to similarly high attack rates and mortality of *D. suzukii* than parasitism by the specialist *G. brasiliensis* in the wild. Even if *T. drosophilae* may not achieve the same biocontrol potential as *G. brasiliensis*, it has already been used in augmentative releases and was found to have significant impact on *D. suzukii* densities, especially when reared on the invasive host prior to release (Gonzalez‐Cabrera et al., 2021, 2023; Rossi Stacconi et al., 2019; Woltering et al., 2019). Some studies found that *T. drosophilae* prefers *D. suzukii* over *D. melanogaster* and other fruit fly species and that the adult parasitoids emerging from *D. suzukii* pupae were larger and more fecund than those emerging from *D. melanogaster* (Esteban‐Santiago et al., 2021; Garcia‐Cancino et al., 2020; Häussling et al., 2021; Woltering et al., 2019; Yi et al., 2020). However, the artifically selected population of *T. drosophila* used in the current experiments prefered to oviposit in *D. melanogaster* and selection for improved performance on *D. suzukii* did not change that (Linder et al., 2022). Development of *T. drosophilae* in *D. suzukii* also takes longer than in *D. melanogaster* (Esteban‐Santiago et al., 2021) and can negatively affect sex ratio (Linder et al., 2022). The opposing findings by studies using different populations could be due to geographic differences in *T. drosophilae* genetic composition and phenotypic traits.

Nevertheless, we show here that the use of laboratory selection to improve the performance of native natural enemies may be a viable alternative or complementary approach for biological control of invasive species. With the approval of *G. brasiliensis* for release in the United States, the focus will likely shift to this specialist parasitoid. Nevertheless, *T. drosophilae* and *G. brasiliensis* will encounter each other in nature and interspecific competition can reduce effectiveness of both (Hougardy et al., 2022). On the other hand, developing a biological control program with multiple available natural enemies can be beneficial as there can be differences in the abundance and performance of different parasitoids locally, regionally, and over time.

## CONFLICT OF INTEREST STATEMENT

The authors declare no conflict of interest.

## Supporting information


Figure S1–S2

## Data Availability

Data for this study are available at: https://doi.org/10.5061/dryad.qnk98sfpm.
