# Supplementary material for: Biological control potential of a laboratory selected generalist parasitoid versus a co‐evolved specialist parasitoid against the invasive Drosophila suzukii
Source: Evol Appl. 2023 Oct 12;16(11):1819–29. doi: 10.1111/eva.13605 (PMC10681468; doi:10.1111/eva.13605)
Supplement: Supplementary file 1 — Figure S1–S2 [file EVA-16-1819-s001.docx]

**Supplementary materials**

Istas and Szucs. 2023. Biological control potential of a laboratory selected generalist parasitoid versus a co-evolved specialist parasitoid against the invasive *Drosophila suzukii*


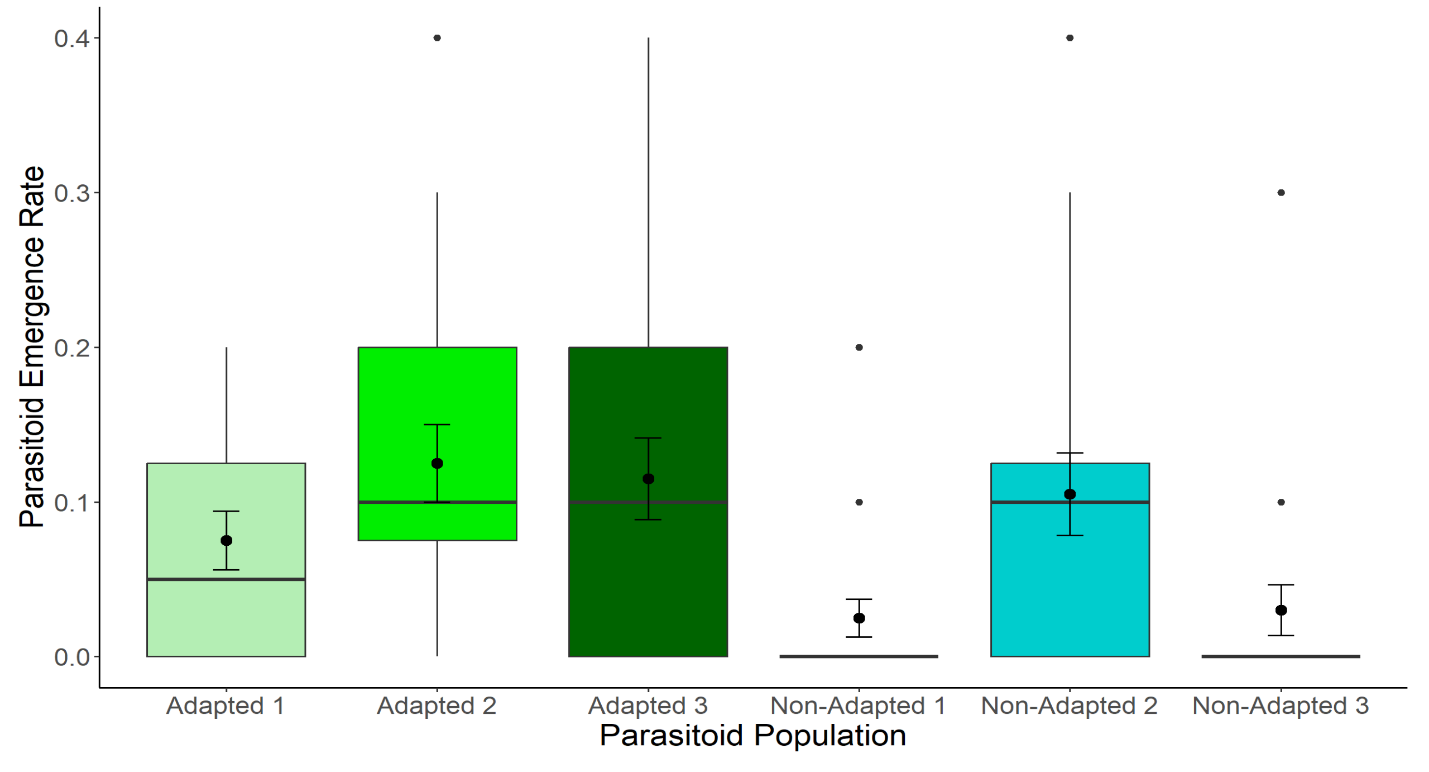


**Figure S1.** The proportion of *Drosophila suzukii* pupae that had successful emergence of *Trichopria drosophilae.* Three replicate populations of *T. drosophilae* had previously been either selected (Adapted 1-3) or had not been selected (Non-Adapted 1-3) for improved developmental success on *D. suzukii.* Dots indicate outlier observations, the horizontal line indicates the median with the box representing the interquartile range, and vertical lines are 1.5 times the interquartile range. Means and standard errors are shown within each box plot.


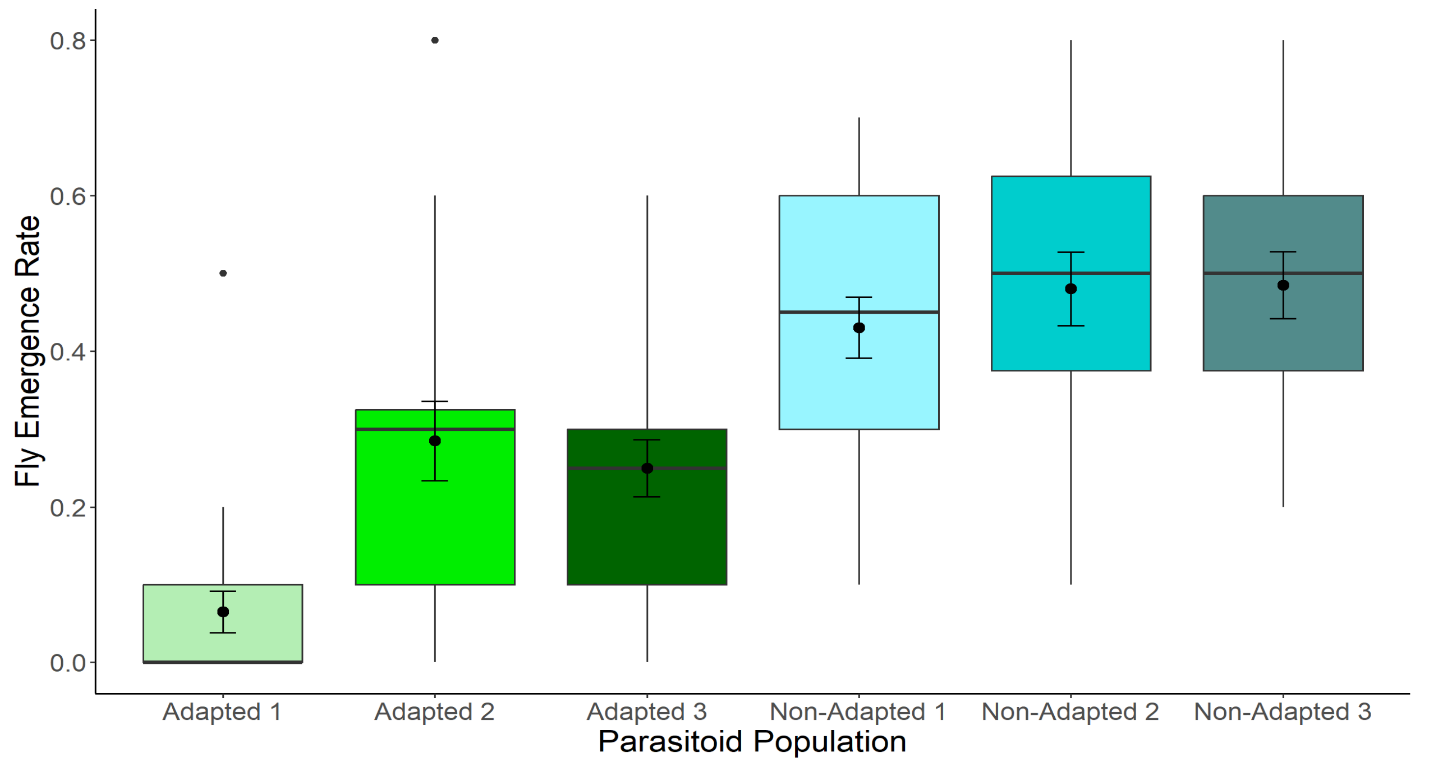


**Figure S2.** The proportion of *Drosophila suzukii* pupae that yielded adult flies when attacked by three replicate populations of *T. drosophilae* that had previously been either selected (Adapted 1-3) or had not been selected (Non-Adapted 1-3) for improved developmental success on *D. suzukii.* Dots indicate outlier observations, the horizontal line indicates the median with the box representing the interquartile range, and vertical lines are 1.5 times the interquartile range. Means and standard errors are shown within each box plot.
